# The Effects of Cognitively Challenging Physical Activity Games versus Health-Related Fitness Activities on Students’ Executive Functions and Situational Interest in Physical Education: A Group-Randomized Controlled Trial

**DOI:** 10.3390/ejihpe13050060

**Published:** 2023-04-26

**Authors:** Athanasios Kolovelonis, Marios Goudas

**Affiliations:** Department of Physical Education and Sport Science, University of Thessaly, 42100 Trikala, Greece; mgoudas@pe.uth.gr

**Keywords:** inhibition, cognitive flexibility, design fluency, cognitive engagement, situational interest

## Abstract

This study compared cognitively challenging physical activity games and health-related fitness activities in terms of their effects on students’ executive functions and situational interest in physical education. A total of 102 fourth- and fifth-grade students (56 boys, 46 girls) participated in this study. A group-randomized controlled trial design involving an acute experiment was used. Two intact classes of students (one fourth-grade and one fifth-grade) were randomly assigned to each one of the three groups. Students in Group 1 participated in cognitively challenging physical activity games, students in Group 2 participated in activities for developing their health-related fitness, and Group 3 students were the control group without physical education. Executive functions were measured pre- and post-intervention with the design fluency test, whereas situational interest was only measured post-intervention with the situational interest scale. Group 1 students who played cognitively challenging physical activity games had increased their executive functions’ scores more than the Group 2 students involved in health-related fitness activities. Students of both these groups outperformed control group students. Moreover, Group 1 students reported higher levels of instant enjoyment and total interest than Group 2 students. The results of this study suggest that cognitively challenging physical activity games can be an effective means for enhancing executive functions, and motivate students to be involved in interesting and enjoyable forms of physical activity.

## 1. Introduction

Participating in regular physical activity is related to important health-related benefits, including physical, mental, and socio-emotional health [1,2] and academic performance [3]. School physical education may play a significant role in enhancing students’ physical activity levels. Traditionally, the enhancement of students’ physical activity within physical education involves simple fitness routines, aerobic exercises, resistance-training or aerobic activities (e.g., running and walking). These kind of activities focus mainly on increasing energy expenditure and promoting health-related outcomes. Recently, however, a new approach on designing physical activity programs has emerged. This view is informed by evidence showing positive relations between participation in physical activity and functional changes in the brain, and suggests that complex motor tasks with increased cognitive demands may facilitate cognitive development [4,5]. This tendency also suggests that physical activity programs should promote energy expenditure while students are involved in challenging conditions [6]. These challenging conditions are present in novel, cognitively complex and unpredictable tasks which require problem solving and mental demands [7]. Focusing both on qualitative (e.g., duration and intensity) and quantitative (e.g., cognitive demands) aspects of physical activity can have beneficial effects on students’ psychomotor, cognitive and physical development [5,8,9]. From this point of view, physical activity programs should adopt a shift “from simply moving to moving with thought” [10], and focus on motor and cognitive development, jointly. However, despite its promising perspectives, the tenets of this approach should be empirically tested.

Executive functions have attracted researchers’ interest in the field of physical and cognitive development [6]. Executive functions are higher-order cognitive processes that are involved in goal-oriented behavior to facilitate flexibility in cognitively demanding situations that require increased levels of attention and concentration [11]. Inhibition, working memory and cognitive flexibility are identified as three core executive functions [10]. Inhibition enables students to respond to changing situations that block and control habitual actions or thoughts, in order to act effectively. For example, a student inhibits an already learned response to act in a way that fits to the new task conditions. Working memory represents the storage of information that is necessary for effective actions during learning or performance (e.g., a student holds in their working memory the instructions for solving a problem). Cognitive flexibility helps students shift their attention among task demands and change their approaches in problem solving or to be flexible when dealing with task demands (e.g., a child uses different strategies to solve a problem when those previously used were ineffective). Executive functions have important implications for health and quality of life [11]. In particular, executive functions are essential for readiness in school [12], academic [13], and sport performance [14,15,16]. They are also involved in learning processes, such as problem solving, planning, decision making, recognizing errors and correcting them [17]. Furthermore, executive functions are related with self-regulated learning [18] governing improvements in students’ self-regulation processes [19]. This way, executive functions can lead to positive outcomes in physical education, such as increased performance, enjoyment, satisfaction and self-efficacy [20,21,22].

Executive functions may be enhanced by physical activity [23]. Indeed, research evidence in the field of physical activity has suggested that executive functions can benefit from long-term interventions [24,25]. Moreover, some systematic reviews and meta-analyses focused on the effectiveness that different types of physical activity programs had on children’s executive functions. In particular, these reviews involved programs emphasizing in the quantity (e.g., high intensity or duration) or the quality (e.g., enriched with cognitive challenges) of physical activity [26], aerobic physical activities or physical activity with cognitive demands [23], and interventions involving aerobic, motor skill, and cognitive challenging tasks [27]. This research showed that the cognitively enriched physical activity programs were those with the larger effect sizes. However, research evidence regarding the potential effects of acute bouts of physical activity on students’ executive functions has generally revealed inconsistent results [28]. Some findings supported that acute bouts of physical activity can have a positive impact on executive functions [29,30]. For example, it was found that an acute exergame-based physical activity session with increased cognitive demands enhanced students’ executive functions (i.e., cognitive flexibility) [31]. In contrast, Bedard et al. [32] reported no effects from an acute session of cognitively engaging physical activity (i.e., children playing a large game of Connect 4) on 6-8-year-old children’s scores in a flanker task (i.e., accuracy and reaction time). Moreover, Egger et al. [33] found that a classroom-based intervention, including breaks with cognitively enriched physical activity, had no effects on updating or inhibition, and had negative effects on shifting. It seems that several factors, including the duration, the intensity and the cognitive engagement of a program, may determine the acute effects of physical activity on executive functions [34]. Indeed, according to recent evidence, not all physical activity types can positively affect executive functions [4]. Therefore, considering the mixed results of previous findings, further evidence regarding the most appropriate types of physical activity for promoting students’ executive functions is warranted. Such information would facilitate the development, as well as the implementation of effective physical activity programs in physical education.

Physical activity games enriched with cognitive challenges may be a favorable type of physical activity for helping students to promote their physical activity and cognitive involvement in physical education settings. These games can be designed to purposely involve students in cognitively demanding and challenging conditions that require problem solving and mental effort [7], and include novel and non-automatized tasks [35]. Three principles of facilitating mental involvement (contextual interference, mental control and discovery) are the basis for designing physical activity games enriched with cognitive challenges and demands [36]. Changing the playing environment, the conditions or the rules of a game and forcing children to select unpredictable movement sequences are effective ways of creating contextual interference. Mental control is developed through different types of games. For example, stopping games require students to react in alternating signals that force them to stop or to go and to override prior actions. Another type of game, the updating game, requires keeping or handling information stored in working memory, whereas in switching games, participants should stop their regular movements to act in a very different way. To promote discovery, students are involved in solving problems that have multiple acceptable solutions by participating in games that allow them to find out how to play and which strategies to use [36].

Very little research has examined the effectiveness of physical activity games that are cognitively challenging. Recently, Kolovelonis and Goudas [37] conducted an acute experiment in physical education to compare three types of these games that were based on the three principles of mental involvement, respectively. All types of these games were effective in triggering children’s executive functions. Kolovelonis and Goudas [38] reported higher scores in an executive functions test for children who played these games, compared to those who were involved in track and field or soccer skills and to control group students. An intervention including cognitively enriched physical activity games in physical education increased students’ executive functions [39]. These findings suggest that these games are a promising means for involving students in cognitively demanding physical activities within physical education.

Another important characteristic of the content chosen for physical education is to attract students’ interest to actively participate in the lesson. Indeed, in a physical education setting, situational interest is considered as a significant motivational variable [40], and increased levels of motivation in physical education are related with higher out-of-school physical activity participation [41]. Furthermore, students’ motivation to participate in physical activities may be increased if movement skills are delivered using a wide range of interesting and enjoyable activities or games [42]. The students’ situational interest can be increased through their involvement in tasks perceived as new and challenging, or tasks offering enjoyment and providing a chance to explore possibilities. For example, elementary students who played exergames reported higher situational interest compared to students who participated in a cardiovascular fitness unit [43]. In previous research, high levels of situational interest for the cognitively challenging physical activity games [37] and higher levels of novelty regarding these games compared to teaching track and field or soccer skills [38] were found.

### The Present Study

The characteristics of the exercise, including quantitative (e.g., intensity, duration) and qualitative (e.g., coordinative or cognitive demands) parameters [4,5] may play a role in the mixed results found regarding the effects of acute bouts of physical activity on executive functions [31,33]; that is, the type of physical activity may be a critical factor for enhancing students’ executive functions [4]. For example, the degree of cognitive involvement in a physical activity session may determine its effects on students’ executive functions [34], such as exposing students to developmentally appropriate quality physical education that involves demanding levels of both physical effort and cognitive involvement, which may positively affect their executive functions [25,44].

Involving students in physically and cognitively enriched physical activity programs may be undertaken through the use of cognitively enriched physical activity games. However, little research has examined the effectiveness of programs including these games. Some respective evidence from research in physical education has shown that these games increased children’s executive functions [37,38]. However, these studies compared these games with physical education sessions including sport skills [38] or with control group students who were not involved in physical education [37]. The current study expanded Kolovelonis and Goudas’ study [37] by combining all three types of these games in a single session. Moreover, the current study replicated and expanded Kolovelonis and Goudas study [38] by comparing these games with a widely used type of program for promoting students’ physical activity within physical education. In particular, the students of the comparison group were involved in a physical education session including activities for enhancing their health-related fitness components (i.e., muscular strength and endurance, cardiovascular endurance and flexibility). This comparison would reveal if cognitively challenging physical activity games can be an equivalent or a better form of physical activity for enhancing children’s executive functions than other forms of physical activity.

These two approaches (cognitively challenging physical activity games versus activities for promoting students’ health-related fitness components) were also compared in terms of their effects on a post-test measure of situational interest, which considers an important motivational variable in physical education [40]. Tasks with different characteristics may have different effects on students’ situational interest. Preliminary evidence has suggested that cognitively enriched physical activity games can attract children’s situational interest [37]. However, further evidence from studies comparing the effects of these games on students’ situational interest with other types of physical activities is warranted. Such information, especially if it comes from research in authentic physical education settings, may help physical educators to design and implement effective programs for promoting their students’ physical activity.

This study aimed to compare a single session of cognitively challenging physical activity games with a session including activities for enhancing students’ health-related fitness components regarding their effects on executive functions. Post-test comparisons between the groups regarding situational interest were also involved. A control group with students who did not participate in physical education was also included. It was expected that students who would be involved in either the cognitively challenging physical activity games or the health-related fitness activities would increase their post-test executive functions scores compared to pre-test, and these improvements would be higher than that of control group students. Moreover, it was expected that cognitively challenging physical activity games would engage students in higher cognitive involvement, and thus would have greater effects on students’ executive functions compared to the health-related fitness program. Furthermore, students who played the games were expected to report increased scores in the dimensions of the situational interest scale compared to students who were involved in health-related fitness activities.

## 2. Materials and Methods

### 2.1. Design

The design of this study involved a group-randomized controlled trial with an acute field experiment in physical education. Three groups of students were involved: (a) Group 1, including students who played cognitively challenging physical activity games; (b) Group 2, including students who participated in a session for developing their health-related fitness components; and (c) Group 3, a control group without physical education. Executive functions were measured pre-test and post-test, and situational interest only post-test.

### 2.2. Participants and Settings

A total of 102 students (Mage = 10.13, SD = 0.57, 56 boys, 46 girls) participated in this study. Only 44 of these students attended 3 fourth-grade classes, and 56 students attended 3 fifth-grade classes of 3 primary schools. Intact classes were involved in the design of this study. In particular, 1 fourth-grade and 1 fifth-grade intact class from each school were randomly assigned to Group 1 (36 students, 18 boys, 18 girls), Group 2 (34 students, 19 boys, 15 girls), and Group 3 (32 students, 19 boys, 13 girls). Students came from a middle socioeconomic status.

Greek physical education is mandatory, coeducational, and delivered by specialized physical education teachers. Fourth-grade students attend three 45 min classes and fifth-grade students attend two 45 min classes per week. The main content of physical education for these grades includes team sports (e.g., soccer, volleyball, basketball), individual sports (e.g., gymnastics and track and field) and dance and rhythm activities. Elementary schools involved in this study were located in a middle-sized city and had outdoor sport facilities for team sports (e.g., basketball and volleyball) and a wide school yard for other sport-related activities.

### 2.3. Measures

#### 2.3.1. The Design Fluency Test

Executive functions were measured by the design fluency test [45]. The advantages of this test are that it is both appealing to children and can be administrated at class level. Three different conditions of the test are included, and a sheet including 25 square boxes with unstructured arrays of dots is available for each one. For each test conditions, four consecutive straight lines should be used for drawing different designs by connecting dots with a pencil in 60 s. In the first condition, five solid dots are included in each box and students should generate novel designs by connecting these dots. In the second condition, five solid and five blank dots are included in each box and students should generate designs by connecting only blank dots. In the third condition, five solid and five blank dots are included in each box and students should generate novel designs by connecting a solid and a blank dot in turn (starting from a blank or a solid dot). The number of correct and unique designs in each test condition represented students’ score in the respective test condition. The first test condition measures design fluency, the second measures inhibition, and the third measures cognitive flexibility. The total score in the design fluency test was also calculated by adding the scores of the three conditions [45].

#### 2.3.2. Situational Interest Scale

This scale [46] consists of five dimensions of situational interest: novelty (e.g., “What we were learning was a new activity for me to do, which I did for the first time”); instant enjoyment (e.g., “What we were learning was appealing/amusing to me”); exploration intention (e.g., “I’d like to know more about how to do what we were learning”); attention demand (e.g., “What we were learning demanded my high attention”); and challenge (e.g., “What we were learning was hard for me to do”). Each dimension consists of three items. An overall situational interest subscale with four items is also included (e.g., “What we were learning attracted me (to participate)”). Students provided their responses using a five-point Likert scale ranging from 1 (strongly disagree) to 5 (strongly agree). The Greek adaptation of the scale [37] demonstrated acceptable psychometric properties showing a good model fit of the sixth-factor solution, χ^2^ (137) = 159.82, *p* = 0.088, χ^2^/df = 1.17, NNFI = 0.980, CFI = 0.984, RMSEA = 0.034 (90% CI: 0.000–0.055). Satisfactory internal consistency (Cronbach’s alpha) in this study was found for instant enjoyment (0.82), novelty (0.94), attention demand (0.78), exploration intention (0.77), challenge (0.68), and total interest (0.89).

### 2.4. Study Procedures

The University Ethics Review Committee and Ministry of Education provided the ethical approval for this study (1522, 5/6/2019). School principals and physical education teachers provided their own permissions while parental written consent was obtained from all students participating voluntarily in this study. An experimenter with a bachelor’s and master’s degree in physical education and extensive experience in delivering interventions in physical education implemented all sessions of this study. The implementation of the session for both the experimental conditions was based on written detailed lesson plans. These plans were tested during a pilot implementation with children not involved in the present study, which resulted in appropriate adjustments. After the completion of each session, the experimenter checked if all the components of the session were implemented appropriately. To facilitate this process, the experimenter was keeping related notes during the implementation of each session. Moreover, after the end of the session, she kept additional notes regarding the children’s involvement and the fidelity of the implementation from each session. All sessions were implemented according to the plan. Group 1 and 2 students completed the executive function test (i.e., design fluency) one week before the experiment. The test was administered by the experimenter in students’ classrooms. Students were provided with appropriate instructions for each test condition. The experimenter demonstrated the completion of each test condition on the classroom’s blackboard. Next, students completed the test. After participating in the acute experiment, Group 1 and 2 students were post-tested in executive functions (i.e., design fluency test) and responded via the situational interest scale. Control group students (Group 3) completed the pre- and post-test measures of the design fluency test one week apart following the same procedures as Group 1 and 2. Between pre- and post-test, to avoid possible confounds of being involved in regular physical education, students of all groups attended a similar number of physical education classes without motor, sport activities or games, and instead focused on the history of modern Olympics and the health-related benefits of physical activity.

### 2.5. Description of the Experimental Conditions

Students in Group 1 participated in a 45 min session including 5 cognitively challenging physical activity games. For designing these games, the three principles of highlighting contextual interference, promoting mental control and discovery were used [36]. In particular, to create contextual interference, the game conditions changed unpredictably requiring from students to react in a different series of actions. In other games, students had to appropriately manipulate information kept in their memory, in order to respond appropriately in alternating signals to override prior actions, or to stop their movements in order to find a different way of acting. Some games focused on promoting discovery by involving students in problem solving, games requiring multiple solutions or the selection of the most effective strategies.

Examples of games included in this session were the hop-pop-tag, a modified version of crazy traffic lights, the mirror and the maps [36]. The hop-pop-tag is a modified tag game based on the principle of contextual interference. In this game, students tried to tag any other student, and at the same time, had to avoid being tagged themselves. Students who were tagged sat on the floor and could return to the game when the student who tagged him/her became tagged. In a modified crazy traffic lights game emphasizing mental control, students performed specific movements or a series of movements responding to verbal instructions, directions or signals provided by the experimenter. Contradictory instructions and signals were also introduced, requiring students to inhibit their routine actions in order to perform correctly. Similarly, the mirror game required students to reproduce the movement actions represented by their teammate, which progressively became longer and more complex. The maps game promoted discovery, requiring students to move around and select and perform a novel sequence of movements.

Group 2 students participated in a 45 min session including activities for developing health-related fitness components. In particular, after an 8 min organization and warm-up, students performed activities for enhancing their muscular endurance (e.g., curl-ups, dorsal raises, modified push-ups, modified dips, semi-squats, and lunges). After that, students practiced in a 9 min section with combined walking and running activities (alternating between 2 min running and 1 min walking), and the session closed with a 7 min flexibility activity.

### 2.6. Statistical Analysis

Considering that entire physical education classes were included in this study, baseline differences between groups including gender and grade as potential differential factors, were examined for the three test conditions, and the total design fluency score through a 3 (Group) X 2 (Grade) X 2 (Gender) MANOVA and ANOVA, respectively. Intervention effects for the first (i.e., fluency), the second condition (i.e., inhibition) and the total design fluency test score, were examined with separate 3 (Group) X 2 (Time) ANOVA with repeated measures followed by pre- to post-test comparisons within each group and post-test comparisons between all of the groups. For cognitive flexibility (i.e., third condition of the design fluency), a one-way ANCOVA was conducted due to pre-test differences between groups, followed by between group comparisons. One-way MANOVA was used to compare groups on their situational interest scores, followed by univariate tests and between group comparisons. The effects sizes (i.e., partial *η*^2^ and Cohen’s *d*) were calculated [47]. Values for Cohen’s *d* below 0.50 represent small effects, values between 0.50 and 0.80 represent medium effects, and values above 0.80 represent large effects. For partial *η*^2^, values below 0.06 represent small effects, values between 0.06 and 0.14 represent medium effects, and values above 0.14 represent large effects.

## 3. Results

### 3.1. Preliminary Analysis

Pre-test and post-test data for all groups regarding design fluency tests were normally distributed, meeting the criterion of less than ±1.96 for skewedness, and kurtosis reflecting normally distributed data [48]. A small deviation was found only in the post-test data regarding cognitive flexibility in Group 3.

Descriptive statistics for students’ scores in executive functions, for all groups of the study, are presented in Table 1. Descriptive statistics for students’ scores in situational interest and correlations among the situational interest subscales are presented in Table 2. A 3 (Group) X 2 (Grade) X 2 (Gender) MANOVA showed a nonsignificant Group X Grade X Gender interaction, *F*(6, 178) = 0.149, *p* = 0.989, in pre-test scores of fluency, inhibition and cognitive flexibility. Moreover, all the two-way interactions and the main effects for gender and grade were nonsignificant. In contrast, a significant main effect for group was found in cognitive flexibility, *F*(2, 90) = 4.47, *p* = 0.014, *η*^2^ = 0.09. Group 2 students had lower pre-test scores in cognitive flexibility compared to Group 1 and Group 3 students. A nonsignificant Group X Grade X Gender interaction, *F*(2, 90) = 0.009, *p* = 0.991, in the pre-test total score in the design fluency test was found. All the two-way interactions and the main effects for gender and grade were also nonsignificant.

### 3.2. Acute Effects on Students’ Executive Functions

#### 3.2.1. Fluency

**Figure 1:**
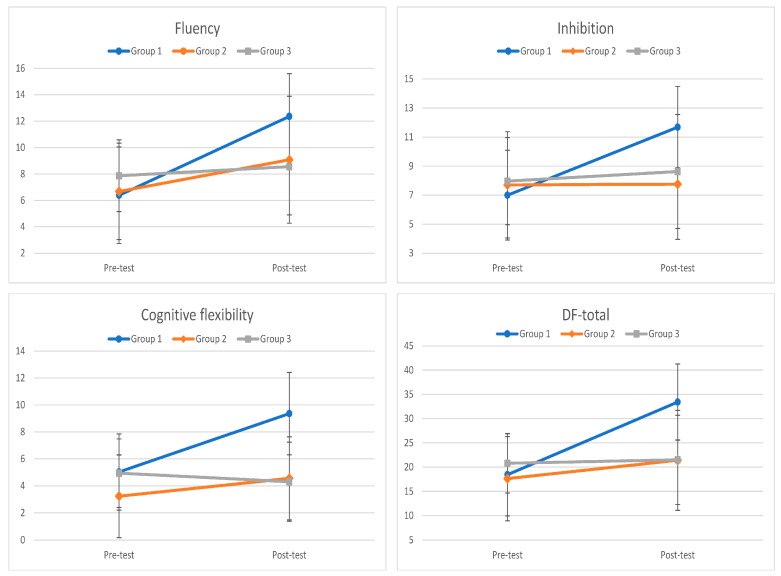
Group and Time interaction in the three conditions (fluency, inhibition, cognitive flexibility) and in the total score of the design fluency test.

The 3 (Group) X 2 (Time) repeated measures ANOVA revealed a significant Group X Time interaction for fluency (Figure 1), *F*(2, 99) = 29.14, *p* < 0.001, *η*^2^ = 0.37. Significant pre- test to post-test improvements were found for Group 1, *t*(35) = −13.38, *p* < 0.001, *d* = 1.73, and Group 2, *t*(33) = −4.17, *p* < 0.001, *d* = 0.56, but not for Group 3, *t*(31) = −1.46, *p* = 0.155, *d* = 0.21. Post-test comparisons between groups showed that Group 1 outperformed Group 2, *t*(68) = 3.35, *p* < 0.001, *d* = 0.80, and Group 3, *t*(66) = 4.54, *p* < 0.001, *d* = 1.10. Nonsignificant differences were found between Groups 2 and 3, *t*(64) = −0.50, *p* = 0.621, *d* = 0.12.

#### 3.2.2. Inhibition

The 3 (Group) X 2 (Time) repeated measures ANOVA revealed a significant Group X Time interaction for inhibition (Figure 1), *F*(2, 99) = 26.57, *p* < 0.001, *η*^2^ = 0.35. A significant pre-test to post-test improvement was found for Group 1, *t*(35) = −11.62, *p* < 0.001, *d* = 1.59, but not for Group 2, *t*(33) = −0.11, *p* = 0.913, *d* = 0.01, and Group 3, *t*(31) = −1.19, *p* = 0.244, *d* = 0.19. Post-test comparisons between groups showed that Group 1 outperformed Group 2, *t*(68) = 4.95, *p* < 0.001, *d* = 1.18, and Group 3, *t*(66) = 3.75, *p* < 0.001, *d* = 0.90. Nonsignificant differences between Groups 2 and 3, *t*(64) = −0.91, *p* = 0.369, *d* = 0.23, were found.

#### 3.2.3. Cognitive Flexibility

The one-way ANCOVA showed that after the adjustment of the pre-test differences, *F*(1, 98) = 82.06, *p* < 0.001, *η*^2^ =0.46, the three groups differed significantly in their post-test scores in cognitive flexibility, *F*(2, 98) = 44.40, *p* < 0.001, *η*^2^ = 0.48. Group 1 had higher scores compared to Group 2, *p* < 0.001, *d* = 1.56, and Group 3, *p* < 0.001, *d* = 1.69, and Group 2 compared to Group 3, *p* = 0.032, *d* = 1.03 (Figure 1).

#### 3.2.4. Total Score in the Design Fluency Test

The 3 (Group) X 2 (Time) repeated measures ANOVA revealed a significant Group X Time interaction for the design fluency total score, *F*(2, 99) = 67.46, *p* < 0.001, *η*^2^ = 0.58 (Figure 1). Significant pre-test to post-test improvements were found for Group 1, *t* (35) = −18.90, *p* < 0.001, *d* = 1.83, and Group 2, *t*(33) = −3.48, *p* < 0.001, *d* = 0.40, but not for Group 3, *t*(31) = −0.83, *p* = 0.412, *d* = 0.09. Post-test comparisons between groups showed that Group 1 outperformed Group 2, *t*(68) = 5.50, *p* < 0.001, *d* = 1.31, and Group 3, *t*(66) = 5.76, *p* < 0.001, *d* = 1.39. Nonsignificant differences between Groups 2 and 3, *t*(64) = −0.04, *p* = 0.971, *d* = 0.01, were found.

### 3.3. Differences on Situational Interest

The one-way MANOVA showed a multivariate effect of Group, *F*(6, 63) = 4.81, *p* < 0.001, *η*^2^ = 0.35, on students’ scores in situational interest subscales. Univariate tests revealed significant Group differences in total interest, *F*(1, 68) = 8.90, *p* = 0.004, *η*^2^ = 0.12, and in instant enjoyment, *F*(1, 68) = 13.87, *p* < 0.001, *η*^2^ = 0.17. In particular, Group 1 students compared to Group 2 students scored higher in total interest (*p* = 0.004, *d* = 0.71) and in instant enjoyment (*p* < 0.001, *d* = 0.89).

## 4. Discussion

Cognitively challenging physical activity games is an approach for enhancing students’ physical activity and involving them in cognitively challenging conditions [6]. This study explored the effectiveness of these games as a means for enhancing students’ executive functions and situational interest. Generally, the findings of the study suggested that these games can positively affect both students’ executive functions and situational interest.

Consistent with our hypothesis, students who played cognitively enriched physical activity games or were involved in the session with health-related fitness activities improved their post-test scores in the executive functions compared to pre-test ones, while the control group did not. These results supported the previous respective evidence in the field of physical education, regarding the beneficial effects of the cognitively enriched physical activity games in promoting children’s executive functions [37,38]. Furthermore, in the present study, the sizes of the effects from these games were generally substantial for students’ executive functions.

Although physical activity may positively affect executive functions, not all types of physical activity are considered equally effective [4]. Research findings are rather mixed regarding the effects of acute bouts of physical activity on students’ executive functions [28]. This study provided evidence that a single session with cognitively challenging physical activity games can have a large acute effect on students’ executive functions. This evidence supported previously reported findings showing that executive functions can be triggered after participating in cognitively enriched physical activity [29,30,31]. Moreover, it supported views that the level of cognitive engagement during physical activity may determine the magnitude of the effects of the acute bouts of physical activity on students’ executive functions [34]. Within this study, the substantial sizes of the effects of the cognitively challenging physical activity games suggested that these games are cognitively enriched and appropriate for triggering students’ executive functions. Such information is important for developing and implementing effective interventions in physical education.

Moreover, consistent with previous evidence [26], inhibition mostly benefited from these games. Indeed, these games involved students in cognitively challenging and demanding conditions that required them to inhibit apparent or routine responses, and to act in a different way, whereas fitness exercises required specific automatized motor responses. Gains in cognitive flexibility were found in both experimental groups, although they were noticeably higher in the group of students who played cognitively challenging physical activity games, rather than in the group with fitness exercises. Involving students in games and tasks requiring motor discovery and production of multiple and novel responses may contribute to these effects. Future research should explore if the cognitively challenging physical activity games have differential effects on the different aspects of executive functions [49]. This research should also include working memory that was not measured in this study.

Most importantly, the present study showed that the cognitively challenging physical activity games had stronger effects on executive functions compared to the respective effects of health-related fitness activities. Consistent with previous evidence [30,50], the students who were involved in the health-related fitness activities improved significantly from pre-test to post-test in their executive functions. However, the size of the effects of the cognitively challenging physical activity games was two or three times higher than the effects of the health-related fitness activities. This means that an appropriately designed physical activity intervention, including physical activity games enriched with cognitive challenges, can significantly impact students’ executive functions. According to a recent review, physical activity programs without cognitive demands had lower effects on executive functions compared to cognitively enriched programs [4].

Theoretical explanations for the acute effects of exercise have been proposed including cognitive, psychological, and physiological mechanisms, as well as the quantitative (e.g., intensity and duration) and qualitative (e.g., cognitive demands) characteristics of the physical exercise [5]. Based on that, the positive effects of the cognitively challenging physical activity games may be attributed to their unique characteristics. The design of these games follows the principles of establishing contextual interference, highlighting mental control and enhancing discovery [36] to create unpredictable and cognitively complex game conditions. Thus, by playing these games, students should respond to the changing game conditions, and react to alternating, and in some cases, contradictory signals, and select appropriate actions using the information stored in their working memory, or provide multiple solutions to a problem. All of these conditions increase the coordinative complexity in the physical tasks and set cognitive challenges to students who are not simply moving during physical education, but are “moving with thought” [10]. Therefore, such games may be considered appropriate for creating optimal task conditions to trigger children’s executive functions in physical education.

The present findings also showed that after playing cognitively challenging physical activity games, students reported higher scores in total interest and in instant enjoyment compared to students who participated in health-related fitness activities. It seems that students enjoyed playing these games more than participating in activities for promoting their health-related fitness. Previous research has also provided preliminary evidence showing high levels of total interest and enjoyment among students after participating in cognitively challenging physical activity games [37], and increased levels of novelty compared to teaching track and field or soccer skills [38]. Increasing students’ situational interest and enjoyment can be considered a significant effect of these games. Selecting appealing and enjoyable activities is a means for enhancing students’ motivation for participating in physical activity during physical education. Indeed, tasks with different characteristics may have different effects on students’ situational interest. For example, playing exergames resulted in higher situational interest compared to a cardiovascular fitness unit [43]. It has also been suggested that students’ motivation in physical education can be increased if movement skills, activities and games are enjoyable, and attract students’ interest to participate [42]. Moreover, fun and enjoyment have been found to be strong motivators for involving adolescents and adults in physical activity [51]. According to students, some of the most important benefits of physical activity, except health-related benefits, were to have fun and feel successful [52].

Enhancing students’ situational interest in physical education can also have a positive impact on their motivation to participate [40], which in turn can positively affect other important outcomes, such as participating in out-of-school physical activity [41]. Cognitively enriched physical activity games can contribute to involving students in intrinsically motivating physical activities and games that provide immediate gratification, enjoyment, and fun. Recent research examining students’ views regarding these games provided such evidence [53]. Most importantly, such interventions may be more effective on enhancing students’ executive functions. Indeed, it has been suggested that making physical activities enjoyable, personally meaningful, and relevant for students is an important factor for increasing the effects of physical activity interventions on students’ executive functions [4].

The present results supported the new approach for designing and implementing cognitively demanding physical activity interventions [6]. Adopting this approach, beneficial effects for students in multiple domains can be pursued [9]. From an applied perspective, choosing physical activity games that are enriched with cognitive demands in physical education programs, can be viewed as an appropriate means for triggering students’ executive functions. Most importantly, students have fun and enjoy their participation in these games. This is significant for physical educators who struggle to increase their student’s motivation for participating in physical activities during physical education. Indeed, there is evidence that some types or forms of physical activity tasks are not so attractive or appealing for some students [54]. Therefore, physical educators should enrich their physical activity programs involving physical activity games with cognitive challenges to make them more appealing and attractive for their students. Furthermore, physical educators may adapt games or fitness activities by adding cognitive challenges and demands to make them more appealing and attractive for their students. This can be done by adding new rules in games, avoiding repetition of automatized movements, changing the conditions of the games, or asking from students to respond to open-ended problems. For example, adding an extra ball in a basketball game will introduce students to novel and cognitive-demanding conditions for responding effectively with this new rule. Moreover, asking students to produce multiple and novel responses in a movement task rather than to move following a movement routine can involve them in a problem-solving process requiring cognitive involvement.

The present study has some limitations. In particular, this study focused on comparing different types of physical activity in terms of their effects on children’s executive functions and situational interest. However, measures of actual physical activity, as well as manipulation checks of physical effort and cognitive engagement during these physical education sessions were not included. Future research may address this limitation by measuring students’ physical activity when participating in the different types of physical activity, and examining potential relations between the amount of physical activity during a session and the development of students’ cognitive functions. Considering that this study compared cognitively challenging physical activity games against a session including health-related fitness activities, further research should also examine the effects of other types for promoting students’ physical activity within physical education (e.g., aerobic routines and different types of dances). These studies should also involve additional socio-motivational measures, such as participation motivation, self-efficacy, attitudes for physical activity, or intentions for participating in out-of-school physical activity. Such research will provide further evidence for the potential effects of the cognitively challenging physical activity games and the benefits of their involvement in physical education practice. Moreover, long-term interventions using retention measures are needed to explore the longitudinal effects of these games on students’ executive functions and motivational-related variables.

## 5. Conclusions

This study suggested that participating in cognitively challenging physical activity games has beneficial effects on children’s executive functions in physical education within schools. Furthermore, students who played these games reported higher scores in total interest and in instant enjoyment compared to students who participated in health-related fitness activities. Thus, these games can be the basis of cognitively enriched physical activity programs in the field of physical education, focusing on promoting students’ physical and cognitive development, jointly.

## Figures and Tables

**Table 1:** Descriptive statistics for executive functions.

	Group 1				Group 2				Group 3			
	Pre-Test		Post-Test		Pre-Test		Post-Test		Pre-Test		Post-Test	
Variable	M	SD	M	SD	M	SD	M	SD	M	SD	M	SD
Fluency	6.39	3.65	12.36	3.24	6.68	3.65	9.08	4.81	7.87	2.72	8.56	3.65
Inhibition	7.00	3.10	11.69	2.80	7.71	3.66	7.76	3.80	7.97	3.00	8.63	3.92
Cognitive flexibility	5.03	2.82	9.36	3.06	3.24	3.05	4.56	3.08	4.94	2.54	4.31	2.93
DF-total	18.42	8.48	33.42	7.86	17.62	8.68	21.41	10.30	20.78	6.11	21.50	9.18

Note: DF: Design Fluency, Group 1: Cognitively challenging physical activity games; Group 2: Developing health-related fitness components; Group 3: Control group.

**Table 2:** Descriptive statistics and correlations for situational interest.

	Group 1		Group 2		Correlations				
	Post-Test		Post-Test						
Variable	M	SD	M	SD	1	2	3	4	5
Total interest	4.06	1.01	3.28	1.18	-				
Instant enjoyment	4.22	0.93	3.25	1.23	0.83 *	-			
Exploration intention	3.54	1.14	3.53	1.35	0.58 *	0.63 *	-		
Attention demand	4.07	0.90	3.75	1.16	0.71 *	0.73 *	0.68 *	-	
Challenge	2.27	1.00	2.35	1.27	0.44 *	0.41 *	0.44 *	0.43 *	-
Novelty	2.85	1.54	2.53	1.59	0.53 *	0.44 *	0.44 *	0.46 *	0.65 *

Note: Group 1: cognitively challenging physical activity games; Group 2: developing health-related fitness components. * Significant correlations (*p* < 0.001).

## Data Availability

The data underlying the results presented in the study are part of a research program and are available on request from the second author (M.G.; mgoudas@pe.uth.gr).
